# Methylation levels at IGF2 and GNAS DMRs in infants born to preeclamptic pregnancies

**DOI:** 10.1186/1471-2164-14-472

**Published:** 2013-07-12

**Authors:** Jing He, Aiping Zhang, Min Fang, Rong Fang, Jiamei Ge, Yuan Jiang, Hong Zhang, Cong Han, Xiaoqun Ye, Danqing Yu, Hefeng Huang, Yun Liu, Minyue Dong

**Affiliations:** 1 Women’s Hospital, School of Medicine, Zhejiang University, Add: 1 Xueshi Road, Hangzhou, Zhejiang Province 310006, China; 2 Bio-X Institutes, Key Laboratory for the Genetics of Developmental and Neuropsychiatric Disorders, Ministry of Education, Shanghai Jiao Tong University, Shanghai, China; 3 Shaoxing Women and Children’s Hospital, Shaoxing, China; 4 Huzhou Maternity and Child Care Hospital, Huzhou, China; 5 Jiaxing Maternity and Child Care Hospital, Jiaxing, China; 6 Ningbo Women and Children’s Hospital, Ningbo, China; 7 Institutes of Biomedical Sciences, Fudan University, Shanghai, China; 8 Department of Biochemistry and Molecular Biology, Key Laboratory of Molecular Medicine, the Ministry of Education, Fudan University Shanghai Medical College, Add: 303 Mingdao Building, 138 Yixueyuan Road, Shanghai 200032, PR China; 9 Key Laboratory of Reproductive Genetics, Ministry of Education, Zhejiang University, Hangzhou, China

**Keywords:** Preeclampsia, Offspring, DNA methylation, IGF2, GNAS, Metabolic diseases

## Abstract

**Background:**

Offspring of pregnancy complicated with preeclampsia are at high risk for hypertension, stroke and possibly obesity. The mechanisms behind the association of intrauterine exposure to preeclampsia and high risk of health problems in the later life remain largely unknown. The aims of the current investigation were to determine the changes in DNA methylation at IGF2 and GNAS DMR in offspring of preeclamptic pregnancy and to explore the possible mechanisms underlying the association between maternal preeclampsia and high risk for health problems in the later life of their offspring.

**Results:**

Umbilical cord blood was taken from infants born to women of preeclampsia (n=56), gestational hypertension (n=23) and normal pregnancy (n=81). DNA methylation levels of IGF2 and GNAS DMR were determined by Massarray quantitative methylation analysis. Methylation levels at IGF2 DMR were significantly lower in preeclampsia than normal pregnancy. The average methylation level at IGF2 DMR was significantly correlated with preeclampsia even after birth weight, maternal age, gestational age at delivery and fetal gender were adjusted. The difference in methylation level was not significantly different between mild and severe preeclampsia. The methylation level at GNAS DMR was not significantly correlated with birth weight, maternal age, gestational age at delivery, fetal gender, preeclampsia or gestational hypertension.

**Conclusions:**

We concluded preeclampsia induced a decrease in methylation level at IGF 2 DMR, and this might be among the mechanisms behind the association between intrauterine exposure to preeclampsia and high risk for metabolic diseases in the later life of the infants.

## Background

Preeclampsia, affecting 2-8% of pregnancies, manifests maternal hypertension and proteinuria in the second half of pregnancy, and is pathologically characterized by shadow implantation of placenta, small placenta and inadequate placental perfusion [1,2]. The disease not only increases maternal and neonatal mortality and morbidity [1,2] but also poses adverse effects on future health of the offspring. The offspring of preelamptic women have increased blood pressure during childhood and adolescence [3,4], pulmonary and systemic vascular dysfunction during adolescence [5], and nearly double the risk of stroke in later life [6]. The effect of intrauterine exposure to preeclampsia on adiposity remains controversial, but some investigations revealed that male offspring of preeclamptic pregnancy had higher BMI, waist circumference, subscapular skinfold thicknesses and body fat percentage than offspring of normal pregnancy [7,8].

Small placenta, low placental perfusion and subsequently under-nutrition and inadequate oxygen supply of the fetus lead to low birth weight of neonates. Preeclampsia caused substantial preterm delivery, which also results in low birth weight [1,2]. Low birth weight is associated with the increased risk of arterial hypertension, carotid arteriosclerosis and mortality caused by coronary heart disease or stroke in adulthood more than 20 years ago [9]. However, some investigations indicate that the association of preeclampsia with health problems in later life of offspring is independent of birth weight [3]. The mechanisms underlying this association attracted the attention of scientists. Alterations in cardiac structure [10], vascular structure and function [11], sympatho-adrenal function [12], renal function [13], immune function and inflammation [14], and endocrine status [15] have been proposed to be involved in the long-term programming of cardio-vascular diseases in the late life of offspring of preeclamptic pregnancies [1,2], however, the molecular mechanism remains largely unknown.

The persistent epigenetic change induced by prenatal environmental conditions in imprinted genes may be among the mechanisms contributing to the association between preeclampsia and late life health in humans. Imprinted genes play important roles in embryonic growth and development as well as in placental function. Epigenetic disruption of imprinted genes due to early exposure to adverse environment was proposed with enhanced susceptibility to adult chronic diseases [16]. Insulin-like growth factor2 (IGF2) is a paternally expressed gene while guanine nucleotide binding protein, alpha stimulating (GNAS) an imprinted gene with a highly complex imprinted expression pattern, which gives rise to maternally, paternally, and biallelically expressed transcripts [17,18]. The methylation at differentially methylated region (DMR) of these imprinted genes is established before gastrulation, and is very sensitive to early developmental environment, but can be relatively stable throughout the course of individual’s life [17,18]. The investigation in subjects conceived during Dutch famine revealed that the exposure to prenatal famine resulted in the persistent difference in DNA methylation of IGF-2, GNAS and other imprinted genes [19,20]. The methylation levels of IGF-2 DMR were affected by folic acid intake before or during pregnancy and depression in pregnancy [21]. The alterations in methylation levels of these imprinted genes regulating growth and metabolism were associated with the low birth weight induced by these poor prenatal conditions and subsequently contribute to the development of diabetes and hypertension in late life [18,22,23]. However, a recent investigation revealed that DNA methylation levels of IGF2, GNAS and leptin were not significantly different between small for gestational age (SGA) and appropriate for gestational age (AGA) [18]. Those findings indicate that there is a need for further investigation on the effect of poor prenatal condition on the DNA methylation of imprinted genes that regulate growth and metabolism.

Herein, to observe the effect of preeclampsia, an important risk factor for low birth weight, on DNA methylation of the fetus and to explore the potential molecular mechanisms linking preeclampsia to the increased risk of cardio-vascular diseases in late life, we analyzed the methylation levels at DMRs of IGF2 and GNAS of umbilical cord blood lymphocytes of neonates born to normal pregnancy, gestational hypertension and preeclampsia. The IGF2 DMR was first evaluated by Cui et al. [24], and was highly correlated with IGF2 expression [25]. The GNAS DMR was maternally methylated and first evaluated by Liu et al. [26], containing an imprinting control element that specifically regulates the imprinting status of GNAS [27]. We found that maternal preeclampsia induced a decrease in DNA methylation level at IGF2 DMR in infants.

## Methods

### Subjects

Fifty-six women of preeclampsia (17 of mild and 39 of severe), 23 women with gestational hypertension and 81 normally pregnant women were recruited in Women’s Hospital, School of Medicine, Zhejiang University, Shaoxing Women and Children’s Hospital, Ningbo Women and Children’s Hospital, Huzhou Maternity and Child Care Hospital and Jiaxing Maternity and Child Care Hospital.

Pregnancy was diagnosed upon positive human chorionic gonadotropin test after missed menstruation. Gestational age was calculated by menstrual dating. Ultrasound was performed to confirm pregnancy and gestational age. Gestational hypertension was defined as following [28]: a systolic blood pressure of 140 mm Hg or higher or a diastolic blood pressure of 90 mm Hg or higher on two occasions at least six hours apart occurring after 20 weeks of gestation in a pregnant woman with previously normal blood pressure and without detectable urinary protein. Preeclampsia were diagnosed and classified according to the criteria recommended by American College of Obstetrics and Gynecologist (ACOG): a systolic blood pressure of 140 mm Hg or higher or a diastolic blood pressure of 90 mm Hg or higher on two occasions at least six hours apart occurring after 20 weeks of gestation in a pregnant woman with previously normal blood pressure and detectable urinary protein (≥1 + by dipstick or 0.3 g/24 h and more) [28]. Severe preeclampsia was defined as a blood pressure greater than or equal to 160/110 mm Hg with either a urine dipstick showing 3+ or 4+ in a random urine sample or greater than 5.0 g of proteinuria over 24 hours [28]. Other evidence of severe disease included elevated serum creatinine, eclampsia, pulmonary edema, oliguria (less than 500 ml per 24 hours), fetal growth restriction, oligohydramnios and symptoms suggesting significant end-organ involvement (headache, visual disturbance, or epigastric or right upper quadrant pain). Women who met criteria of preeclampsia but not severe preeclampsia were diagnosed mild preeclampsia.

Exclusion criteria were multiple gestation, diabetes mellitus, chronic hypertension, infectious diseases recognized in pregnancy, premature rupture of membrane, active labor, polyhydramnios and signs of other concurrent medical complication. The control women had no sign of gestational complications and fetal distress and gave birth to healthy neonates of appropriate size for gestational age.

Clinical data and demographic data were collected according to the medical records. The approval of the current study was obtained from Institutional Ethical committee of Women’s Hospital, School of Medicine, Zhejiang University, and all the participants provided their informed consents.

### DNA methylation analysis

Umbilical cord blood samples were collected in Ethylene Diamine Tetraacetic Acid (EDTA)-treated tubes at delivery. Lymphocytes of infants were isolated and stored at -80C until use. Total DNA was isolated from lymphocytes using buffer ATL, proteinase K, and RNase A (Qiagen, Inc., Valencia, CA) followed by phenol–chloroform extraction and ethanol precipitation. Bisulfite conversion of DNA was carried out using the Epitect Bisulfite Kit (Qiagen Inc., Valencia, CA).

Quantitative methylation analysis of DNA was performed using MassARRAY EpiTYPER assays (Sequenom, San Diego, CA). Primers were designed using Epidesigner (Sequenom, San Diego, CA; http://www.epidesigner.com) to cover the CG-rich regions with amplicons in a target range of 400–600 bp. Each reverse primer was designed to contain a T7 promoter sequence tag (5'-CAG TAA TAC GAC TCA CTA TAG GGA GAA GGC T-3') for in vitro transcription, and each forward primer incorporated a 10-mer tag (5'-AGG AAG AGA G-3') to balance the primer annealing temperature with that of the primer containing the T7 tag. Polymerase chain reaction (PCR) amplification was performed using HotStarTaq (Qiagen, Inc, Valencia, CA) with the following parameters: polymerase activation at 95°C for 5 min, followed by denaturation at 94°C for 20 sec, annealing at 60°C for 25 sec, and extension at 72°C for 1 min for a total of 40 cycles, with a final incubation at 72°C for 5 min. After dephosphorylation of unincorporated dNTPs, the processed poly-chain reaction (PCR) products were used in in vitro transcription reactions (T-cleavage assay) according to the manufacturer’s standard protocol (Sequenom, San Diego, CA). The transcription products were conditioned to remove bilvalent cation adducts by dilution with 20 μl H2O and addition of 6 mg of Clean Resin (Sequenom, San Diego, CA). The samples were then spotted on a 384-pad Spectro-CHIP (Sequenom, San Diego, CA) using a MassARRAY Nanodispenser (Samsung, Irvine, CA), followed by spectral acquisition on a MassARRAY analyzer compact MALDI-TOF MS (Sequenom, San Diego, CA). Fragments containing CpG sites were analyzed with EpiTyper software (Sequenom, San Diego, CA) to generate quantitative methylation fractions at these sites. CpGs unites that yielded data in greater than 90% of the samples passed the initial quality control. Poor–quality data for the qualitative methylation of each CpGs unit were excluded. Duplicate units which inhibit the display of all duplicate CpG ratios were also excluded from the data analysis. Methylation was measured at 6 CpG dinucleotides at the IGF2 DMR in Chr11: 2169100–2169551, version 2009 (GRCh37/hg19), CpG site 3: 2,169,499; CpG site 4: 2,169,400; CpG site 6: 2,169,371; CpG site 7: 2,169,290; CpG site 9: 2,169,175; CpG site 10: 2,169,138. Methylation was measured at 8 CpG dinucleotides at the GNAS DMR in Chr 20: 57,415,713-57,416,072, version 2009 (GRCh37/hg19), CpG site 2: 57,415,774; CpG site 4: 57,415,808; CpG site 5: 57,415,838; CpG site 7: 57,415,889; CpG site 8: 57,415,908; CpG site 9: 57,415,956; CpG site 12: 57,416,034.

### Statistical analysis

The Kolmogorov-Smirnov tests were used to evaluate the distribution of data. Student t-tests were used for the comparison of continuous data between groups while one-way ANOVA as well as Bonferroni test for the multiple-group comparison. Chi-square test was used for the analysis of categorical data. Linear mixed model analysis was used for the relationship of methylation level with disease, birth weight, maternal age, gestational age at delivery and fetal gender. Linear regression was used to evaluate the correlation of methylation level with birth weight in normal pregnancy, gestational hypertension, or preeclampsia respectively. SPSS statistical package (Statistical Analysis System, Chicago, IL) was used for the data analysis. Values of P<0.05 were considered to be statistically significant.

## Results

As shown in Table 1, there was significant difference in maternal age, gestational age at delivery and neonatal birth weight among normal pregnancy, gestational hypertension and preeclampsia. Gestational age at delivery was significantly shorter in preeclampsia than normal pregnancy and gestational hypertension (P<0.001 for both), but was not significantly different between normal pregnancy and gestational hypertension. The differences in neonatal birth weight were significant between normal pregnancy and gestational hypertension (P=0.033), between normal pregnancy and preeclampsia (P<0.001) and between gestational hypertension and preeclampsia (P<0.001). There was no significant difference in fetal gender among three groups.

**Table 1:** Clinical data and average methylation levels at IGF2 and GNAS DMRs

	**Normal pregnancy**	**Gestational hypertension**	**Preeclampsia**		
N	81	23	56		
Maternal age (y)	28.6±3.4	31.8±4.7	30.8±5.0	F=6.687	P=0.002
Gestational age at delivery (w)	38.70±1.37	38.29±1.90	34.07±3.29	F=72.509	P<0.001
Birth weight (g)	3288±395	3629±543	2473±818	F=45.591	P<0.001
Fetal gender	Male:41	Male:14	Male:30	*X*^2^=0.763	P=0.683
	Female:40	Female:9	Female: 26		
Average methylation level at IGF2 DMR	0.4609±0.0434	0.4526±0.0340	0.4425±0.0415	F=3.242	P=0.042
Average methylation level at GNAS DMR	0.5060±0.0559	0.5087±0.0523	0.5151±0.0530	F=0.473	P=0.624

**Figure 1:**
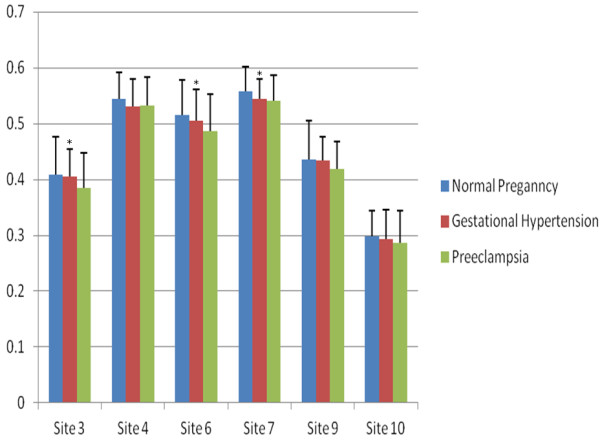
**The comparison of Site-specific methylation levels at IGF2 DMR among normal pregnancy, gestational hypertension and preeclampsia.** There were significant differences in methylation levels (Y-axle) of site 3, 6 and 7 (*: P<0.05 for all) but not the site 4, 9 and 10 among three groups (P>0.05 for all). The methylation levels of site 3, 6 and 7 were significantly lower in preeclampsia than normal pregnancy (P=0.045, 0.009, 0.048, respectively), but not significantly different between normal pregnancy and gestational hypertension, or between gestational hypertension and preeclamspsia (P>0.05 for all).

**Figure 2:**
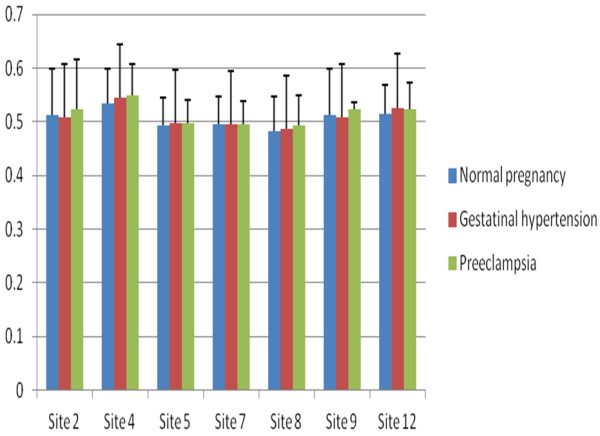
**The comparison of Site-specific methylation levels at GNAS DMR among normal pregnancy, gestational hypertension and preeclampsia.** There were no significant differences in methylation levels (Y-axle) of any site among three groups (P>0.05 for all).

The methylation levels of six CpG sites of IGF2 DMRs and seven CpG sites of GNAS DMRs were detected (Figures 1 and 2). The methylation levels at sites 3, 6 and 7 of IGF2 DMR were significantly lower in preeclampsia than normal pregnancy (P=0.045, 0.009 and 0.048, respectively), but the methylation levels at site 4, 9 and 10 did not significantly differ (P>0.05 for all). However, we did not find any significant differences in methylation level at any sites of IGF2 DMR between normal pregnancy and gestational hypertension or between gestational hypertension and preeclampsia (P>0.05 for all). There were no any significant differences in the methylation levels at 7 sites of GNAS DMR among normal pregnancy, gestational hypertension and preeclampsia (P>0.05 for all). Furthermore, the methylation levels of IGF2 and DNAS DMRs were not significantly different between mild and severe preeclampsia (P>0.05 for all) (data not shown).

The average methylation level at the IGF2 DMR was significantly different among normal pregnancy, gestational hypertension and preeclampsia (F=3.242, P=0.042) (Table 1). The methylation level was significantly lower in preeclampsia than normal pregnancy (P=0.036), but was not significantly different between normal pregnancy and gestational hypertension, or between gestational hypertension and preeclampsia (P>0.05 for both). There was no significant difference in methylation level between mild (0.4464±0.0493) and severe preeclampsia (0.4407±0.0382) (t=0.466, P=0.643). Average methylation level was significantly correlated with preeclampsia even after birth weight, maternal age, gestational age at delivery and fetal gender were adjusted (P<0.05).

The average methylation level at the GNAS DMR was not significantly different among normal pregnancy, gestational hypertension and preeclampsia (F=0.47, P=0.624) (Table 1). There was no significant difference in average methylation level between mild (0.5046±0.0340) and severe preeclampsia (0.5191±0.0586)(t=0.928, P=0.358). The methylation level of GNAS DMR was not significantly correlated with preeclampsia, gestational hypertension, birth weight, maternal age, gestational age at delivery or fetal gender.

## Discussion

In the current investigation, we revealed for the first time that maternal preeclampsia but not gestational hypertension induced a decrease in DNA methylation level at IGF2 DMR in infants. Since aberrant methylation at IGF2 and subsequently the expression of IGF2 affects postnatal growth and contribute to the development of diabetes, hypertension and other metabolic disorders in late life, our findings implies that preeclampsia-induced decrease in fetal DNA methylation at IGF2 DMR might be among the mechanisms associating maternal preeclampsia and metabolic disorders in late life of the offspring.

Early embryo development is a crucial period for establishing and maintaining epigenetic marks that are susceptible to nutritional condition of very early stage of mammalian development [29]. Animal studies have indicated that environmental conditions during conception produce persistent changes in epigenetic marks that have life-long phenotypic consequence [23]. Heijmans et al. [19] first described the relevance of periconceptional exposure to under-nutrition and hypomethylation at IGF2 DMR by reporting that maternal exposure to famine during Dutch Winter Hunger of 1944–45 led to hypomethylation at IGF2 DMR in their offspring and proposed that hypomethylation at IGF2 DMR was one of the mechanisms linking low birth weight and high risk of diabetes, hypertension and other metabolic diseases. Hoyo et al. and his colleagues [30] found that depressed mode during pregnancy was associated with 3-fold increase in the risk of low birth weight and low birth weight infants had 2.4% lower methylation at IGF2 DMR compared with infants with normal birth weight. These findings from natural models indicate that poor prenatal conditions, either under-nutrition or maternal disease, induces epigenetic modifications of imprinted genes. In the current study, we observed a decrease in methylation level at IGF2 DMR in cord blood cells of infants from preeclamptic women compared with those born to normally pregnant women, implying that hypoperfusion to fetal-placental unit occurs in very early life of embryonic development and that decreased methylation at IGF2 MDR is among the mechanisms linking exposure to maternal preeclampsia and health problems of late life.

IGF2 is one of the major regulators for embryonic development and fetal growth and its disturbance is involved in fetal growth restriction. However, the relationship between circulating IGF2 and birth weight remains controversial. Kajantie et al. [31] observed that IGF2 level in umbilical cord plasma from preterm infants (born before 32 weeks of gestation) was negatively correlated with birth weight SD score (expression of birth weight adjusted for gestational age), while Hoyo et al. [32] reported that elevated IGF2 level in cord blood was associated with higher birth weight. IGF2 DMR methylation levels control circulating IGF2 concentration. Hoyo et al. observed that lower IGF2 DMR methylation was associated with higher plasma IGF2 protein concentration [32]. In this study, we found that methylation level at IGF2 DMR was negatively correlated with birth weight in normal pregnancy but not in preeclampsia or gestational hypertension. These findings imply that IGF2 regulates fetal growth but the regulatory pattern is disturbed in pathological pregnancies.

The effect of complicated pregnancies on the fetal DNA methylation has been investigated so far. Low birth weight is a characteristic of preeclampsia. To explore the relationship between small for gestational age (SGA), a proxy for intrauterine growth restriction, and DNA methylation levels of imprinted genes, Tobi et al. [18] determined DNA methylation levels at IGF2, GNAS and other imprinted genes in SGA infants born preterm (before 32 weeks of gestation) and compared with infants appropriate for gestational age (AGA) born preterm, and found no significant differences in DNA methylation levels at any imprinted genes detected between AGA infants and SGA infants. Tabano et al. [33] reported that mean methylation values of umbilical cord blood cells resembled peripheral blood cells from normal individuals and mean methylation values of umbilical cord blood cells were similar in SGA (n=5) and AGA cases (n=10). Our findings showed that DNA methylation at IGF2 DMR was lower in infants born to preeclamptic women than those born to controls. However, Tobi et al. [18] found that preeclampsia was not significantly associated with DNA methylation levels at any of these imprinted genes including IGF2 and GNAS in a SGA study with relatively small sample size (n=25). These findings indicate that the effect pattern on fetal epigenetic programming is different in preeclampsia from fetal growth restriction.

The epigenetic modification of GNAS is sensitive to environmental condition and the function of GNAS is associated with diabetes, hypertension and other metabolic diseases. The literature regarding the effect of prenatal condition on the methylation at GNAS DMR is limited. Tobi et al. [18] reported that the methylation level at GNAS DMR was not significantly different between SGA and AGA individuals. Similarly, we observed comparable methylation levels in infants born to normal pregnancy and preeclampsia. Relatively low birth weight is one of the characteristics of preeclampsia. These limited data indicate that under-nutrition at early life does not affect DNA methylation at GNAS DMR and the methylation level may not be associated with preeclampsia-induced risk of metabolic syndrome.

The current investigation is a cross-sectional design, not a prospective one from early pregnancy onward. This limited the extrapolation of our results. The effect of confounding factors including gene polymorphism on the association observed could not be completely excluded.

## Conclusion

In summary, we observed that maternal preeclampsia induced a decrease in methylation level at IGF-2 DMR compared with normal pregnancy and DNA methylation level at IGF-2 DMR was significantly associated with neonatal birth weight in normal pregnancy but not in preeclampsia or gestational hypertension. Our findings indicated that IGF-2 is one of the mechanisms involved in regulating fetal growth but this regulation was perturbed in preeclampsia and that preeclampsia-induced decrease in fetal DNA methylation at IGF-2 DMR might be among the mechanisms associating maternal preeclampsia and metabolic disorders in late life of the offspring, however, the confounding effect of other factors could not be completely excluded.

## Abbreviations

IGF-2: insulin-like growth factor-2; GNAS: guanine nucleotide binding protein, alpha stimulating; DMR: differentially methylated region; DNA: Deoxyribonucleicacid; EDTA: Ethylene Diamine Tetraacetic Acid; SGA: small for gestational age; AGA: appropriate for gestational age; ACOG: American College of Obstetrics and Gynecology; EDTA: Ethylene Diamine Tetraacetic Acid; PCR: poly-chain reaction.

## Competing interests

The authors declare that they have no competing interests.

## Authors’ contributions

JH (Jing He) participated in experiment design, data analysis and draft of the manuscript. AZ (Aiping Zhang) participated the experiment, data analysis and drafted and revised the manuscript. MF (Min Fang) participated in obtain, analysis and interpretation of data. RF (Rong Fang) participated in obtain, analysis and interpretation of data. JM (Jiamei Ge) participated in obtain, analysis and interpretation of data. YJ (Yuan Jiang) participated in obtain, analysis and interpretation of data. HZ (Hong Zhang) carried out quantitative assay of DNA methylation and data anlysis. CH (Cong Han) participated sample collection, experiment and data analysis. XY (Xiaoqun Ye) participated sample collection, experiment and data analysis. DY (Danqing Yu) participated sample collection, experiment data analysis and draft of manuscript. HH (Hefeng Huang) conceived the study, participated its design and criticized the manuscript. YL (Yun Liu) conceived the study and design, and drafted and revised the manuscript. MD (Minyue Dong) conceived the study and design, coordinated the whole procedures and drafted and revised manuscript. All authors read and approved the final manuscript.
